# A Ray-Tracing-Based Single-Site Localization Method for Non-Line-of-Sight Environments

**DOI:** 10.3390/s24247925

**Published:** 2024-12-11

**Authors:** Shuo Hu, Lixin Guo, Zhongyu Liu

**Affiliations:** School of Physics, Xidian University, Xi’an 710071, China; shu@stu.xidian.edu.cn (S.H.); liuzy@xidian.edu.cn (Z.L.)

**Keywords:** AOA, NLOS, single-site localization, propagation, RT, IRLS

## Abstract

Localization accuracy in non-line-of-sight (NLOS) scenarios is often hindered by the complex nature of multipath propagation. Traditional approaches typically focus on NLOS node identification and error mitigation techniques. However, the intricacies of NLOS localization are intrinsically tied to propagation challenges. In this paper, we propose a novel single-site localization method tailored for complex multipath NLOS environments, leveraging only angle-of-arrival (AOA) estimates in conjunction with a ray-tracing (RT) algorithm. The method transforms NLOS paths into equivalent line-of-sight (LOS) paths through the generation of generalized sources (GSs) via ray tracing. A novel weighting mechanism for GSs is introduced, which, when combined with an iteratively reweighted least squares (IRLS) estimator, significantly improves the localization accuracy of non-cooperative target sources. Furthermore, a multipath similarity displacement matrix (MSDM) is incorporated to enhance accuracy in regions with pronounced multipath fluctuations. Simulation results validate the efficacy of the proposed algorithm, achieving localization performance that approaches the Cramér–Rao lower bound (CRLB), even in challenging NLOS scenarios.

## 1. Introduction

Traditional localization methods assume a LOS condition between the user and the localization station [1]. However, in complex urban environments, there are often intricate NLOS conditions [2]. Obstacles in the LOS path cause reflections, diffractions [3], and transmissions [4] in signal propagation. In such NLOS scenarios, the observed time of arrival (TOA) [5], time difference of arrival (TDOA) [6], AOA [7], and received signal strength (RSS) [8] can introduce significant errors, leading to increased inaccuracies in traditional localization methods [9].

Current research on NLOS localization methods primarily focuses on two aspects: NLOS identification techniques and NLOS error mitigation techniques [10,11,12]. NLOS error identification primarily uses empirical measurement data as support, often applied in multi-sensor cooperative localization [13,14,15,16]. Channel information such as RSS, root mean square delay, and root mean square angle spread is used to identify LOS or NLOS nodes [17,18,19,20,21,22]. Some studies also mention using deep learning tools as a key to identifying localization errors. For instance, [23] employed ultra-wideband (UWB) devices and integrated deep learning to develop a Deep Q-Learning energy-optimized LOS/NLOS framework, effectively improving the localization accuracy of TDOA algorithms in multi-station scenarios. In [24], the author utilized an AdaBoost network classifier to identify NLOS nodes, enhancing the TOA localization accuracy. Additionally, Kalman filtering methods have been used in some studies to filter out NLOS information [25,26,27]. However, in severe NLOS conditions, the LOS path may not be obtainable, leading to degraded localization accuracy for methods relying on LOS path identification [28]. The second approach is NLOS error mitigation techniques. Existing error mitigation methods mainly rely on data bias or noise deviations in known NLOS environments, which limits the applicability of the algorithms in different NLOS scenarios. In [29], the author assumed that all paths were NLOS and introduced a TOA-RSS combined NLOS bias parameter. This method transforms the original non-convex problem into a generalized trust region sub-problem (GTRS) framework, effectively improving localization in NLOS scenarios. In [30], the author employed an equality-constrained Taylor series robust least squares method to suppress NLOS errors in UWB localization systems. This approach effectively reduces the algorithm’s dependence on prior NLOS data. The work in [31] employed a nonlinear weighted least squares method to eliminate NLOS errors in TOA and RSS hybrid localization. By using a convex hull constraint, this method does not require prior NLOS statistical information. The work in [32] proposed a regulation term least-square-based semidefinite programming (RTLS-SDP) method that improves TOA localization accuracy without prior NLOS information. However, these studies address mixed LOS/NLOS scenarios, and the localization performance of these algorithms degrades in purely NLOS conditions.

Additionally, some researchers have focused on using NLOS information for localization. Chee Kiat Seow proposed a method that utilizes single-scattering paths to fuse AOA and TOA for localization [33]. In this approach, multiple-scattering paths are identified and removed, which reduces the impact of complex NLOS paths on localization. However, this method does not address localization challenges in severe NLOS environments. In [34], the author first introduced the concept of virtual stations (VSs) and developed the T two-step weighted least squares (TSWLS) method by incorporating TOA observations. This approach achieved localization in simple geometric NLOS scenarios. The work in [35,36,37] proposed a VS localization method based on geometric maps. This method converts NLOS paths into LOS paths and establishes a joint TSWLS equation for TOA and AOA, effectively reducing the impact of some NLOS paths on localization. However, when localization relies solely on low-order or single-reflection paths, the accuracy is poor in complex multipath scenarios, and clock synchronization errors among multiple stations can further impact localization precision. In [38], the author introduces a system of nonlinear equations by establishing a VS and combining AOA and TDOA estimation parameters. The TSWLS method is applied to achieve single-station localization, effectively mitigating the errors caused by clock synchronization in TDOA localization. However, this approach considers only basic geometric information and overlooks the characteristics of electromagnetic wave propagation channels. Consequently, its localization performance is significantly limited in complex NLOS environments.

To address the limitations of traditional localization algorithms in NLOS scenarios, this paper integrates RT algorithms [39,40] with conventional AOA localization algorithms to develop the ray-tracing localization-based service (RT-LBS) method. This approach effectively enables the localization of non-cooperative target sources (NCTSs) in urban environments using only a single station. Simulations and experimental results demonstrate that the proposed algorithm achieves the CRLB.

In summary, this paper makes the following contributions:

- To improve the performance of existing RT methods and better align them with the proposed localization approach, this paper integrates the advantages of both RT and the shooting and bouncing ray (SBR) technique. Furthermore, an innovative adaptive ray tube structure is introduced, allowing the propagation effects within the environment to be more accurately captured and reflected in the localization algorithm.
- The localization method integrates AOA localization with RT to construct nonlinear equations using GSs generated by sensors in the environment. A heuristic approach for determining equation weights based on angle and power residuals is constructed, and the IRLS method is applied for the precise localization of NCTSs. Simulations and experimental results show that the proposed method reaches the CRLB.
- In NLOS scenarios, regions with rapid multipath birth and death processes can severely compromise the robustness of localization algorithms. To address this, this paper introduces an MSDM designed based on multipath similarity. By integrating the MSDM, the robustness of the localization algorithm in these challenging regions is significantly improved.
- In this paper, the localization algorithm requires repeated invocations of the ray-tracing (RT) process for path generation, which involves extensive traversal of the node tree structure constructed by the RT algorithm. This results in decreased computational efficiency. To address this issue, a fast GPU-based algorithm is proposed, which accelerates the path generation component of the RT within the localization algorithm, thereby significantly improving its overall efficiency.

The rest of this paper is organized as follows: Section 2 introduces improvements to the ray-tracing algorithm. Section 3 illustrates the construction process of the RT-LBS method. Section 4 showcases the relevant simulations and experimental results supporting the fundamental theory of RT-LBS. Section 5 discusses the findings and limitations of this study. Section 6 provides conclusions and future research plans.

## 2. The Improvement of the Ray-Tracing Algorithm

**Figure 1:**
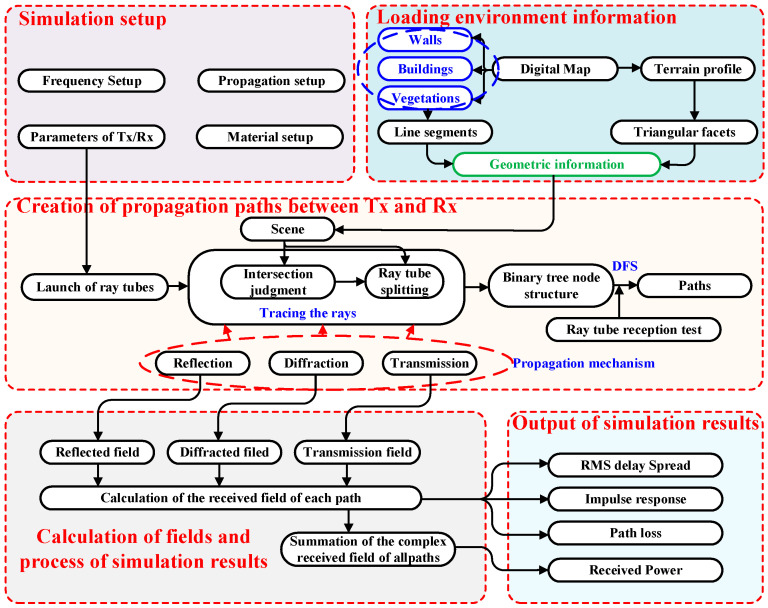
A flowchart of the proposed RT algorithm.

RT methods can be divided into two main categories: the image-based ray-tracing method (IM-RT) [41] and the shooting and bouncing ray method (SBR) [42]. The IM-RT algorithm first constructs a mirror source tree structure within the environment, followed by solving the path to the receiving point. In contrast, the SBR method emits rays uniformly in all directions from the transmitting point and calculates energy paths by employing a receiving sphere. Due to the differing mechanisms of path generation, the accuracy of electromagnetic field calculations using IM-RT is generally higher than that of SBRs. The 2.5D RT method proposed in this paper combines the advantages of IM-RT and SBRs. In Figure 1, a simplified flow diagram of the implementation of the RT model is presented. The corresponding process can be divided into the following main steps: simulation setup, the loading and preprocessing of environmental information, the creation of propagation paths between the Tx and Rx, the calculation of fields and the processing of simulation results, and the output of simulation results.

**Figure 2:**
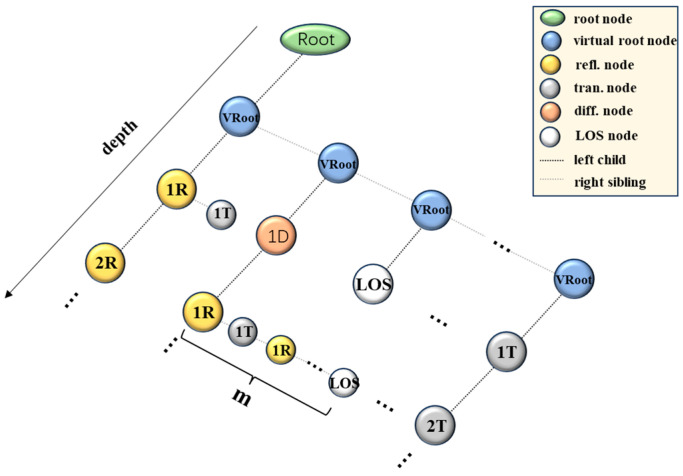
Binary tree structure of ray nodes.

The following section provides a detailed explanation of the key components of the proposed algorithm, including the ray-splitting algorithm, ray reception algorithm, and electromagnetic field computation. First, the overall algorithm adopts the ray tube launching method, where ray tubes are uniformly launched in a two-dimensional plane, and each ray tube is then traced as it undergoes reflection, transmission, diffraction, and other propagation phenomena in the environment. To further improve accuracy, the proposed algorithm employs ray tube reception instead of the receiving sphere as the reception determination criterion. Finally, the coarse path to the receiving point is refined using the IM method to obtain an accurate path. As shown in Figure 2, the propagation of rays results in a binary tree structure of ray nodes [43].

### 2.1. Ray-Splitting Algorithm

**Figure 3:**
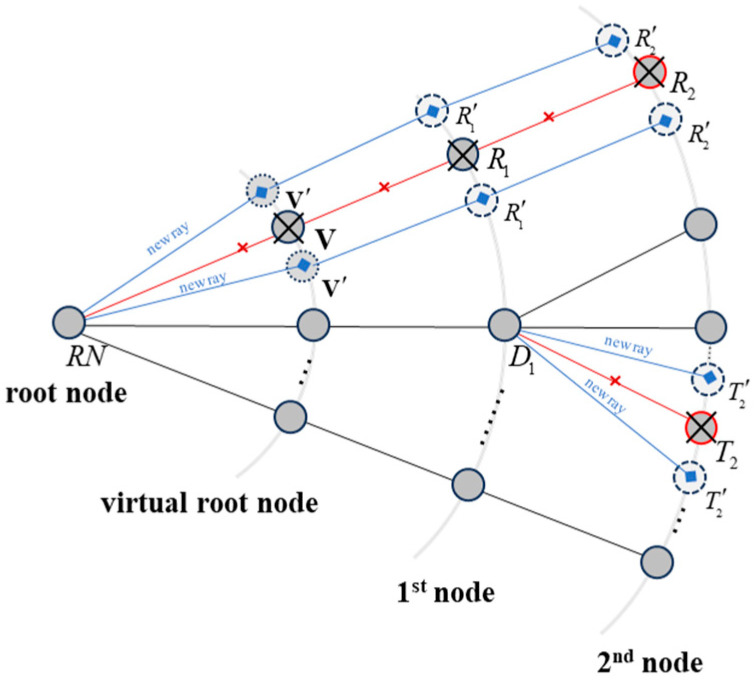
Schematic diagram of ray-splitting structure. Red nodes indicate split nodes that will be deleted, while blue nodes represent newly generated split nodes.

The 2.5D RT algorithm proposed in this paper combines the advantages of the ray tube method and the image method. In the algorithm, rays are designed in the form of ray tubes with splitting attributes. During the initial path search, the uniform splitting threshold of the ray tubes is set based on the size of the geometric facets in the environment (in 2D, this corresponds to the length of line segments). When rays intersect with the environment, the algorithm computes the number of ray splits and generates the corresponding splitting data accordingly. The GS nodes of the split rays are then divided into multiple split GS nodes according to the splitting information. Finally, the algorithm places the split GS nodes into a simulation stack for iterative calculations until all rays are fully traced. Figure 3 presents a schematic diagram of the ray-splitting algorithm, while Algorithm 1 provides the corresponding steps in pseudocode. The coordinates of a GS node $p_{GS}$ are determined by backtracking the propagation distance l from the intersection point $p_{it}$ (reflection, transmission, diffraction, etc.) between the ray and the environment in the opposite direction of the outgoing ray $\vec{r_{o}}$.

(1) $$p_{GS}=p_{it}-l\cdot \vec{r_{o}}$$

**Algorithm 1.** Ray-splitting algorithm**Input:**      Ray-splitting threshold t **Precondition:** Create a stack object stack and stack the virtual root node.**While** stack is not empty  Pop up the top element node ns on the stack  Obtain ray r in ns.   Calculate the intersection point between r and the scene  Calculate the ray-splitting information ξ  **if** ξ>t    Obtain the GS node $g_{ns}$ of ns     Calculate the set of splitting nodes $\left\{g_{i}\right\}$ based on ξ    Mount all nodes in $\left\{g_{i}\right\}$ onto the right sibling node of ns    **delete**
ns  **End if**  Trace rays and execute other programs
**End While**


### 2.2. Ray Reception Algorithm

**Figure 4:**
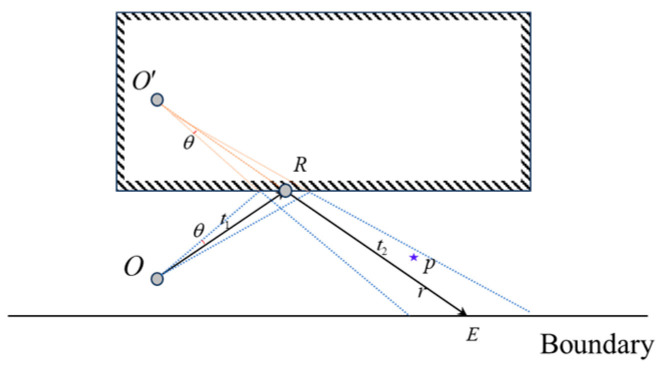
Schematic diagram of ray tube determination and reception. Red lines represent virtual ray tubes, while blue lines indicate the edge rays of the ray tube.

In the SBR method, the determination of ray reception is based on the concept of a receiving sphere. Due to the uneven distribution of rays in space, it is necessary to dynamically adjust the radius of the receiving sphere to achieve appropriate energy reception. The adaptive splitting ray tube in this algorithm effectively avoids the limitations of the receiving sphere mechanism. As shown in Figure 4, when determining whether point p is received by ray tube r, a GS $O^{\prime }$ is constructed at point R relative to O. If the condition in Equation (2) is satisfied, it can be concluded that point p is captured by ray tube r.

(2) $$\left\{\begin{array}{l} t_{1}\leq \left\|\vec{O^{\prime }P}\right\|\leq t_{1}+t_{2}\\ \arccos\left(\frac{\vec{O^{\prime }P}\cdot \vec{O^{\prime }E}}{\left\|\vec{O^{\prime }P}\cdot \vec{O^{\prime }E}\right\|}\right)\leq \theta \end{array}\right.$$

### 2.3. Electromagnetic Field Computation

Once the binary tree structure of the ray nodes is established, a depth-first search (DFS) traversal is conducted on the ray tree, yielding multiple potential paths from the transmitter (Tx) to the receiver (Rx). If there is a LOS condition between the Tx and Rx, the radiation field at arrival point p is calculated according to Equation (3):

(3) $${\vec{E}}_{Los}={\vec{E}}_{0}\frac{e^{-jkr_{0}}}{r_{0}},$$

where ${\vec{E}}_{0}$ denotes the electric field strength at the position of the Tx antenna, k stands for the wave number, and $r_{0}$ is the total length of the LOS propagation path.

In the case where NLOS exists between the Tx and Rx, the LOS field ${\vec{E}}_{0}$ is first computed using Equation (4), and then the contributions of other electromagnetic propagation mechanisms to the field strength, ${\vec{E}}_{inc}$, are progressively calculated along the ray path.

(4) $${\vec{E}}_{NLOS}={\vec{E}}_{inc}\cdot \overset{n}{\underset{h=1}{\prod }}\bar{\bar{R_{h}}}\cdot \overset{m}{\underset{i=1}{\prod }}\bar{\bar{D_{i}}}\cdot \overset{l}{\underset{t=1}{\prod }}\bar{\bar{T_{t}}}\cdot \overset{n+m+l}{\underset{s=1}{\prod }}A_{s}\cdot \overset{n+m+l}{\underset{q=1}{\prod }}e^{-jkr_{q}},$$

where n represents the number of reflections, m represents the number of diffractions, and t represents the number of transmissions along the path. $A_{s}$ is the spherical wave spreading factor, and $r_{q}$ is the distance between the q-th node and the (q + 1)-th node.

The total field strength at the receiving point is obtained by combining the field contributions from all paths and is the sum of the field strengths of all rays. It should be noted that this paper presents two modes for combining the field strengths: $E_{total}$, which is the scalar combination of fields, and ${\vec{E}}_{total}$, which is the vector combination of fields.

(5) $$\left\{\begin{array}{c} E_{total}=\sum_{i=1}^{l}\left|\vec{E_{i}}\right|\\ {\vec{E}}_{total}=\sum_{i=1}^{l}\vec{E_{i}} \end{array}\right.,$$

where l denotes the number of ray paths.

After deriving the field strength formula, by incorporating the Tx antenna gain $G_{t}$, the receiver antenna gain $G_{r}$, and the antenna radiated power $P_{t}$, the received power $P_{r}$ at the field point can be obtained as follows:

(6) $$\begin{array}{cc} P_{r}=10\mathrm{lg}\left(P_{tx}G_{tx}G_{rx}{\left(\frac{\lambda }{4\pi }\right)}^{2}{\left|\frac{E_{total}}{E_{0}}\right|}^{2}\right)+30 & dBm \end{array},$$

## 3. Single-Site Localization Algorithm for NLOS Environment

**Figure 5:**
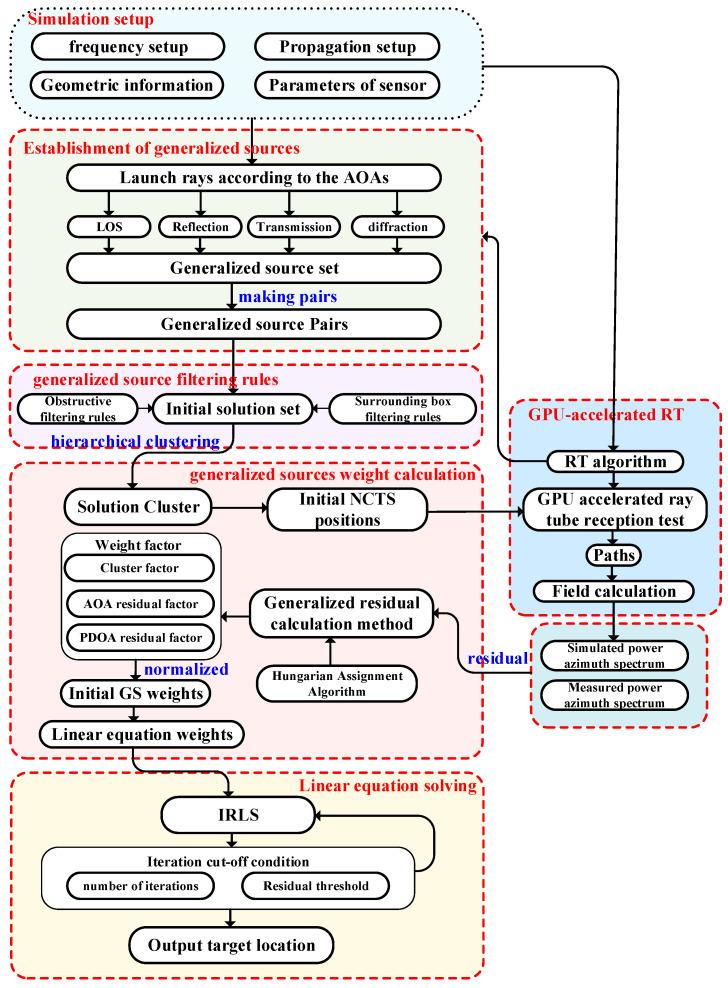
An overview of the overall technical roadmap of the RT-LBS algorithm.

The RT-LBS method proposed in this paper is designed for localization in complex geometrically obstructed environments. This work effectively utilizes the RT method’s (see Section 2) predictive capabilities in complex scenarios, extending traditional AOA localization algorithms. The proposed algorithm enables accurate NLOS localization with a single station. A simplified flowchart of the RT-LBS algorithm is provided in Figure 5. The associated process can be divided into the following key steps: establishment of generalized sources, generalized source filtering rules, generalized source weight calculation, and linear equation solving.

### 3.1. Establishment of Generalized Sources

The RT algorithm can track each ray emitted from the source, thus covering most of the geometric surface features within the environment. In the localization phase, the sensor is treated as a virtual source. This sensor is assumed to receive a set of AOAs $\left\{\phi_{1},\phi_{2},\ldots ,\phi_{n}\right\}$ corresponding to the electromagnetic waves emitted by an NCTS, where n represents the number of angles received by the sensor. Furthermore, it is assumed that the angle measurement error of the sensor follows a zero-mean Gaussian distribution, with the standard deviation $\sigma_{\phi }$. Therefore, each angle interval received by the sensor is $\left[\phi_{i}-\sigma_{\phi },\phi_{i}+\sigma_{\phi }\right]$. Next, ray tubes are uniformly emitted within the angle interval at increments of Δϕ. The algorithm tracks each ray tube and constructs a binary node tree structure. By traversing each node of the binary tree structure, all GSs related to the sensor are obtained.

The position of the reflected GS, GSr, is the location of the mirror image of source S in the geometric environment. The position of the diffracted GS, GSd, is located on the diffraction wedge that generates the diffracted rays. The position of the transmitted GS, GSt, is obtained by backtracking the propagation distance, t, from the transmission node location along the outgoing transmission direction. It is important to note that the involved reflected, transmitted, and diffracted GSs are not limited to first-order. They can undergo multiple reflections, transmissions, and diffractions, forming higher-order GSs through combinations of these interactions. By traversing all possible propagation path nodes in the environment, all GSs related to S are obtained. This allows the corresponding AOA localization equations to be formulated as follows:

(7) $$\left\{\begin{array}{c} \left(x-x_{1}\right)\tan\phi_{1}=y-y_{1}\\ \left(x-x_{2}\right)\tan\phi_{2}=y-y_{2}\\ \vdots \\ \left(x-x_{m}\right)\tan\phi_{m}=y-y_{m} \end{array}\right.,$$

where m denotes the number of the GS, and ϕ stands for the AOA received by the GS.

### 3.2. Generalized Source Filtering Rules

The RT algorithm generates a large number of GSs, each carrying angle information about the NCTS, which can form m localization equations based on the AOA. There is exactly one valid GS in each set of GSs for a given AOA that is in a LOS environment with the NCTS (here, we assume that there is only one multipath arriving at the receiving sensor per angle).

Therefore, the system of linear equations formed by the GS set contains a large amount of noise, making the system ill conditioned, which in turn causes the target localization solution to fail to converge. To address this issue, this paper proposes a filtering method specifically designed for the GS set. This method effectively eliminates invalid equations, improving the stability and accuracy of the localization solution.

In the first step, by selecting two GSs from the GS set and combining them, $Pair_{i,j}=\left\{GS_{i},GS_{j}\right\},i\neq j$, a system of equations can be constructed based on the two selected GSs:

(8) $$\left\{\begin{array}{c} \left(x-x_{i}\right)\tan\phi_{i}=y-y_{i}\\ \left(x-x_{j}\right)\tan\phi_{j}=y-y_{j} \end{array}\right..$$

This equation has an analytical solution, denoted by $P_{i,j}=\left(x_{p},y_{p}\right)$:

(9) $$\left\{\begin{array}{c} x_{p}=\frac{\left(x_{i}\sin\phi_{i}-y_{i}\cos\phi_{i}\right)\cos\phi_{j}-\left(x_{j}\sin\phi_{j}-y_{j}\cos\phi_{j}\right)\cos\phi_{i}}{\sin\phi_{j}}\\ y_{p}=\frac{\left(x_{i}\sin\phi_{i}-y_{i}\cos\phi_{i}\right)\sin\phi_{j}-\left(x_{j}\sin\phi_{j}-y_{j}\cos\phi_{j}\right)\sin\phi_{i}}{\sin\phi_{j}} \end{array}\right..$$

The GS filtering rules proposed in this paper are as follows:

1. If $P_{i,j}$ is located inside a building or outside the solution domain, the position is considered invalid.
2. Construct line segments $s_{1}$ and $s_{2}$ connecting position $P_{i,j}$ with $GS_{i}$ and $GS_{j}$ at the intersection points $node_{i}$ and $node_{j}$, respectively. If $s_{1}$ and $s_{2}$ intersect within the environment, then $P_{i,j}$ is considered an invalid solution.

In the second step, begin looping through the $C_{m}^{2}$ pairs of GSs, solving for the validity of each GS pair. If $Pair_{i,j}$ is valid, increment the count weight w for the corresponding GSs by 1. After traversing all GSs, delete those with a count weight w=0.

Additionally, this paper employs a hash mapping method to eliminate duplicate GSs with overlapping positions. During the hash mapping process, angle data for the identified duplicate GSs are averaged. However, GSs generated by diffraction and transmission nodes are not subject to this duplication removal.

### 3.3. Generalized Source Weight Calculation

Let the remaining number of GSs be l. By combining these remaining sources, $C_{l}^{2}$ pairs of GS combinations are generated, with the solution for each pair denoted by $p_{i,j}$. The solution set $\left\{p_{i,j}\right\}$ from the $C_{l}^{2}$ combinations is then clustered based on a given inter-cluster distance threshold d, resulting in q clusters. The center position of each cluster is denoted by $p_{i},i\in \left[1,q\right]$.

In this study, a generalized residual calculation method is proposed. The method first calculates the path from $p_{i,j}$ to the sensor S and then, using electromagnetic computation techniques, determines the angles and power of the $n_{1}$ multipath components arriving at S. These angles and power values are subsequently sorted in descending order based on power magnitude. Since the power of the NCTS is unknown, it is not feasible to directly compute the residuals based on absolute power values. To address this problem, a received signal strength difference (RSSD)-based residual calculation method is proposed in this study. The original RSS at the sensor is denoted by $pr_{0}$, while the calculated RSS at the sensor from the NCTS is denoted by $pr_{1}$. The RSSD is then computed, and combined with Equations (4) and (6), we can obtain

(10) $$\begin{array}{l} \Delta pr=pr_{1}-pr_{0}\\ =10\mathrm{lg}\left(P_{tx}G_{tx}G_{rx}{\left(\frac{\lambda }{4\pi }\right)}^{2}{\left|\frac{E_{total}^{\left(1\right)}}{E_{0}}\right|}^{2}\right)+30-\left(10\mathrm{lg}\left(P_{tx}G_{tx}G_{rx}{\left(\frac{\lambda }{4\pi }\right)}^{2}{\left|\frac{E_{total}^{\left(0\right)}}{E_{0}}\right|}^{2}\right)+30\right)\\ =20\mathrm{lg}\left(\frac{E_{total}^{\left(1\right)}}{E_{total}^{\left(0\right)}}\right)\\ =20\mathrm{lg}\left(\frac{\sum \overset{n_{1}}{\underset{h=1}{\prod }}\bar{\bar{R_{h}}}\cdot \overset{m_{1}}{\underset{i=1}{\prod }}\bar{\bar{D_{i}}}\cdot \overset{l_{1}}{\underset{t=1}{\prod }}\bar{\bar{T_{t}}}\cdot \overset{n_{1}+m_{1}+l_{1}}{\underset{s=1}{\prod }}A_{s}\cdot \overset{n_{1}+m_{1}+l_{1}}{\underset{q=1}{\prod }}e^{jkr_{q}}}{\sum \overset{n_{0}}{\underset{h=1}{\prod }}\bar{\bar{R_{h}}}\cdot \overset{m_{0}}{\underset{i=1}{\prod }}\bar{\bar{D_{i}}}\cdot \overset{l_{0}}{\underset{t=1}{\prod }}\bar{\bar{T_{t}}}\cdot \overset{n_{0}+m_{0}+l_{0}}{\underset{s=1}{\prod }}A_{s}\cdot \overset{n_{0}+m_{0}+l_{0}}{\underset{q=1}{\prod }}e^{jkr_{q}}}\right) \end{array}.$$

The definitions of the variables in the equation can be found in Equations (4) and (6). From Equation (10), it can be inferred that Δpr is solely related to the number and coefficients of reflections, diffractions, and transmissions between the two paths and is independent of the initial field. Therefore, Δpr can be effectively used to quantify the residual between two positions.

Therefore, after obtaining the original angle–power data $\left\{\phi_{raw,i},\Delta pr_{raw,i}\right\},i\in \left[1,n\right]$ and the simulated angle–power data $\left\{\phi_{sim,i},\Delta pr_{sim,i}\right\},i\in \left[1,n_{1}\right]$, it is often the case that the numbers of multipath components in the measured and simulated data are not identical, i.e., $n_{1}\neq n$. As a result, it is not possible to directly compute the residuals. This paper proposes a generalized residual computation method, which first employs the Hungarian algorithm (as provided by the GLPK open-source library) to determine the minimum residual $\arg\min\left\{\phi_{raw,i},\phi_{sim,j}\right\}$ between two sets of angle data. This process yields the angular residual $r_{\phi }$ and the corresponding assignment sequence. Using the assignment sequence, the power difference residual $r_{\Delta pr}$ is calculated. In cases where $n_{1}<n$, the method also records the number $k=n-n_{1}$ of unmatched original data points.

(11) $$\left\{\begin{array}{l} r_{\phi }=\sum_{i=1}^{\min(n,n_{1})}{\left\|\phi_{raw,i}-\phi_{sim,j}\right\|}_{2}\\ r_{\Delta pr}=\sum_{i=1}^{\min(n,n_{1})}{\left\|\Delta pr_{raw,i}-\Delta pr_{sim,j}\right\|}_{2} \end{array}\right.$$

After calculating the angular residual $r_{\phi ,i}$ and the power difference residual $r_{\Delta p,i}$ for all clusters, the maximum values $r_{\phi ,\max}$ and $r_{\Delta p,\max}$ as well as the average values ${\bar{r}}_{\phi }$ and ${\bar{r}}_{\Delta p}$ of the residuals are determined. Given that instances of unmatched points arise during the residual calculation process, the total residual needs to be adjusted by adding k times the average residual, as expressed by the following equation:

(12) $$\left\{\begin{array}{c} r_{\phi ,i}=r_{\phi ,i}+k{\bar{r}}_{\phi }\\ r_{\Delta p,i}=r_{\Delta p,i}+k{\bar{r}}_{\Delta p} \end{array}\right..$$

Subsequently, the residuals for the cluster are normalized based on the maximum values of residuals $r_{\phi ,\max}$ and $r_{\Delta p,\max}$, yielding the normalized residuals ${\hat{r}}_{\phi ,i}$ and ${\hat{r}}_{\Delta p,i}$. In addition, the size of the cluster is incorporated into the weight calculation, with the normalized weight of the cluster denoted by ${\hat{w}}_{cluster,i}$. The weight expression is then derived as follows:

(13) $$w_{i}=a\frac{1}{{\hat{r}}_{\phi ,i}+\varepsilon }+b\frac{1}{{\hat{r}}_{\Delta p,i}+\varepsilon }+c{\hat{w}}_{cluster,i},$$

where a, b, and c represent the coefficients of the AOA weight, RSSD difference weight, and cluster size weight, respectively. These coefficients must satisfy the condition a+b+c=1, which is typically set to the same weight. ε is a small decimal value, typically set to 1 × 10^−6^.

After determining the weight of each cluster, the weights of the individual GSs within the clusters are updated, yielding a system of linear equations with the assigned initial weights.

### 3.4. IRLS

After determining the weights for all GSs, an IRLS method based on the initial weights is proposed to solve the system of linear equations. The AOA localization equation can be reformulated in terms of residuals as follows:

(14) $$r_{i}\left(x,y\right)=\left(x-x_{i}\right)\tan\phi_{i}-\left(y-y_{i}\right).$$

The objective function $S\left(x,y\right)$ to be optimized in the IRLS method is the weighted sum of squared residuals, which is the squared ℓ2-norm of the residuals, weighted as follows:

(15) $$S\left(x,y\right)=W{\left\|r\right\|}_{2}^{2}=\sum_{i=1}^{m}w_{i}{\left(r_{i}\left(x,y\right)\right)}^{2},$$

where $w_{i}$ denotes the weight of each equation.

In the IRLS iteration process, the initial position $\left(x_{0},y_{0}\right)$ of the NCTS is first estimated. During each subsequent iteration, the residual $r_{i}\left(x_{i},y_{i}\right)$ for each equation is calculated, and the weights are updated accordingly:

(16) $$w_{i+1}=\frac{1}{{\left(r_{i}\left(x_{i},y_{i}\right)\right)}^{2}+\varepsilon },$$

The estimated position after the k-th iteration can be obtained based on the least squares method as follows:

(17) $$\left[\begin{array}{c} x_{i+1}\\ y_{i+1} \end{array}\right]=\left[\begin{array}{c} x_{i}\\ y_{i} \end{array}\right]-{\left(J^{T}WJ\right)}^{-1}J^{T}Wr,$$

where J represents the Jacobian matrix of the residual equation, and r denotes the residual vector.

Additionally, an error threshold must be established. Typically, the iteration stopping criteria can be defined as follows:

1. The value of the objective function $S\left(x,y\right)$ falls below the predefined threshold $s_{a}$;
2. The Euclidean distance between consecutive iterative solutions is less than the threshold $l_{a}$;
3. The number of iterations has reached the maximum limit k.

### 3.5. Cramér–Rao Lower Bound

The CRLB is widely used to evaluate the theoretical limit of localization accuracy under the influence of unbiased noise. It defines the lower bound of localization error in the presence of noise. For the AOA localization method, it is assumed that the measurement angle errors follow a zero-mean Gaussian distribution.

(18) $$\phi_{i}=\tan^{-1}\left(\frac{y-x_{i}}{x-y_{i}}\right)+\eta_{i},$$

where $\eta_{i}$ represents the error term.

The Jacobian matrix is obtained by taking the partial derivatives with respect to x and y:

(19) $$J_{AOA}=\left[\begin{array}{cc} \frac{\partial \phi_{1}}{\partial x} & \frac{\partial \phi_{1}}{\partial y}\\ \frac{\partial \phi_{2}}{\partial x} & \frac{\partial \phi_{2}}{\partial y}\\ \vdots & \vdots \\ \frac{\partial \phi_{m}}{\partial x} & \frac{\partial \phi_{m}}{\partial y} \end{array}\right].$$

In accordance with the definition of the Fisher Information Matrix (FIM), the following expression can be derived:

(20) $$I\left(x\right)=\frac{1}{\sigma^{2}}J^{T}J.$$

Thus, the CRLB is given by the inverse of the FIM, expressed as

(21) $$CRLB\left(x\right)=I{\left(x\right)}^{-1}.$$

## 4. Simulation and Experimental Results

### 4.1. Measurement Campaign

#### 4.1.1. Measurement Equipment

**Figure 6:**
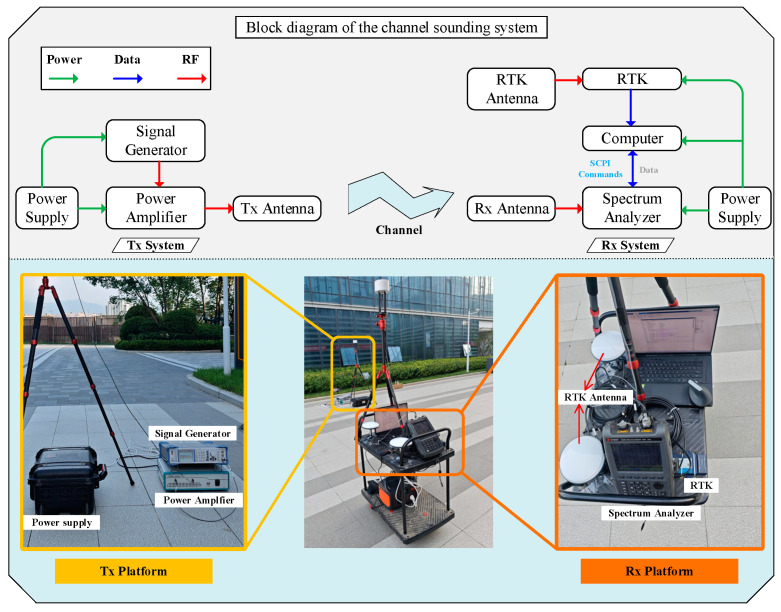
Power measurement system architecture and key equipment. The **upper half** of the figure is the block diagram of the channel sounder used in this paper. The **lower half** is the key equipment of the sounder, including the signal generator, power amplifier, spectrum analyzer, power supplier, RTK, and antennas.

The architecture of the power measurement system and the key instrumentation are illustrated in Figure 6. A continuous-wave (CW) signal is generated by the signal generator and amplified up to a maximum of 43 dBm by the signal amplifier. On the Rx platform, the signal is directly fed from the receiving antenna into a spectrum analyzer, which is controlled by a computer to capture power information. Additionally, a real-time kinematic (RTK) system and its antenna are mounted on a cart to collect real-time positional data. Both the transmitting and receiving antennas are vertically polarized omnidirectional antennas, each with a height of 1.85 m.

**Figure 7:**
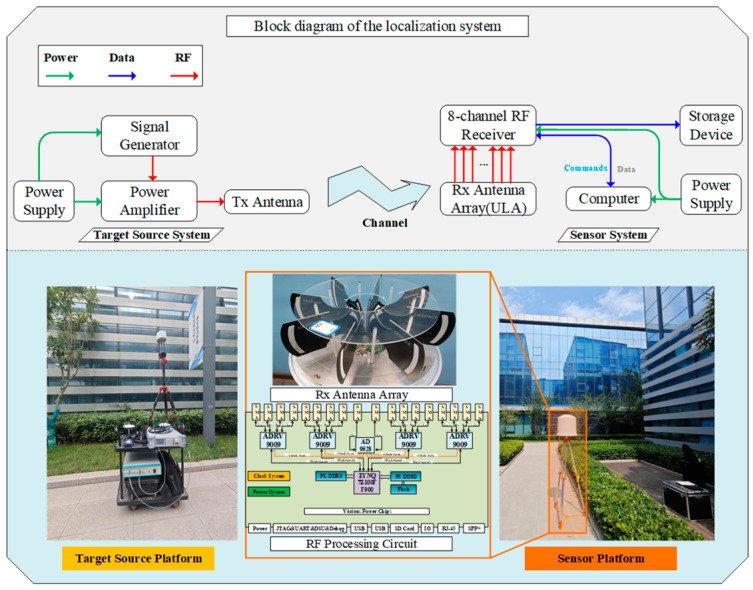
Localization test system architecture and key equipment. The **upper half** of the figure is the block diagram of the localization test system used in this paper. The **lower half** is the key equipment in the signal transmitter system, UCA direction-finding equipment, the Rx antenna array, and the RF processing circuit.

The architecture of the localization test system and the key instruments are shown in Figure 7. The NCTS platform uses the same architecture as the transmission platform in the power measurement system. An eight-channel uniform circular array (UCA) antenna platform is employed to measure the AOA of incoming signals, and the multiple signal classification (MUSIC) algorithm is used to estimate the AOA. Figure 7 presents an image of the eight-channel circular array antenna along with the schematic of the corresponding RF board. Four ADRV9008 chips are used to form the eight-channel receiving system, with clock synchronization achieved through the AD9528 chip. Under anechoic chamber measurement conditions, the direction-finding accuracy can reach approximately 1°.

#### 4.1.2. Measurement Scenarios

**Figure 8:**
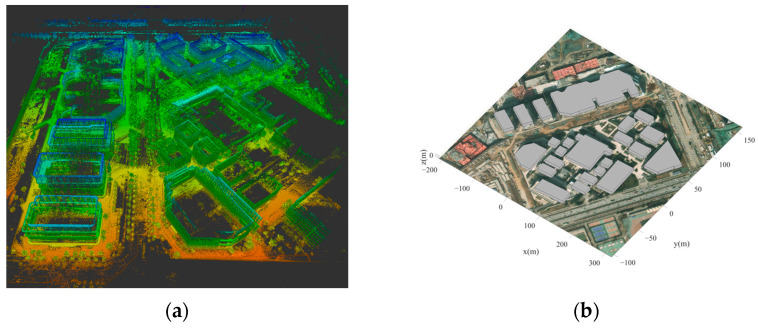
Measurement scenario. (**a**) The raw point cloud image of the measurement scenario. (**b**) The geometric building model extracted from the point cloud.

The measurements were conducted in Xi’an, Shaanxi Province, China, within a modern technology park comprising 23 buildings. The area spans 0.2 km^2^. A geometric model of the technology park was generated using a handheld LiDAR device, which offers an accuracy of up to 5 cm. Figure 8 presents the raw laser point cloud data alongside the reconstructed site floor plan. All measurement scenarios are situated within an urban environment, with the scenarios in this study divided into fading measurement scenarios and localization test scenarios.

During the mobile power measurement process, it is essential to maintain a constant receiver speed of 5 km/h. The measurement duration for each frequency point ranges from approximately 450 to 500 s, with the longest measurement path extending up to 700 m. The street canyons in the measurement area are notably narrow, varying between 6 and 12 m in width. Throughout the measurement campaign, most of the receiver’s path is obstructed by buildings, resulting in NLOS conditions. As the path progresses, the prevalence of these NLOS conditions increases, leading to a substantial rise in propagation losses.

### 4.2. Processing Measured Results and Verification of Models

#### 4.2.1. Power Measurement Data Processing

**Figure 9:**
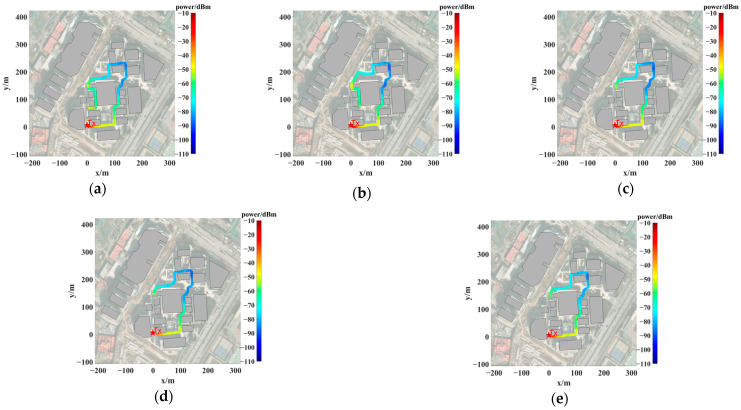
Measurement path and power distribution at (**a**) 3 GHz frequency, (**b**) 3.6 GHz frequency, (**c**) 4 GHz frequency, (**d**) 5 GHz frequency, and (**e**) 5.9 GHz frequency.

A total of five frequency points were measured at 3 GHz, 3.6 GHz, 4 GHz, and 5 GHz during the power measurement process. Figure 9 illustrates the measurement paths for each frequency point, along with the RSS at each location, depicted using a pseudo-color map. The Tx was positioned within a street canyon, and most of the receiver’s path remained in NLOS conditions relative to the Tx. The detailed measurement parameters are provided in Table 1. It is important to note that a fully automated measurement methodology was adopted, with the RTK system and spectrum analyzer recording real-time positional information (with centimeter-level accuracy) and power at a rate of 20 frames per second. The location and power data were synchronized by matching the timestamps of the positional records with those of the power measurements.

**Figure 10:**
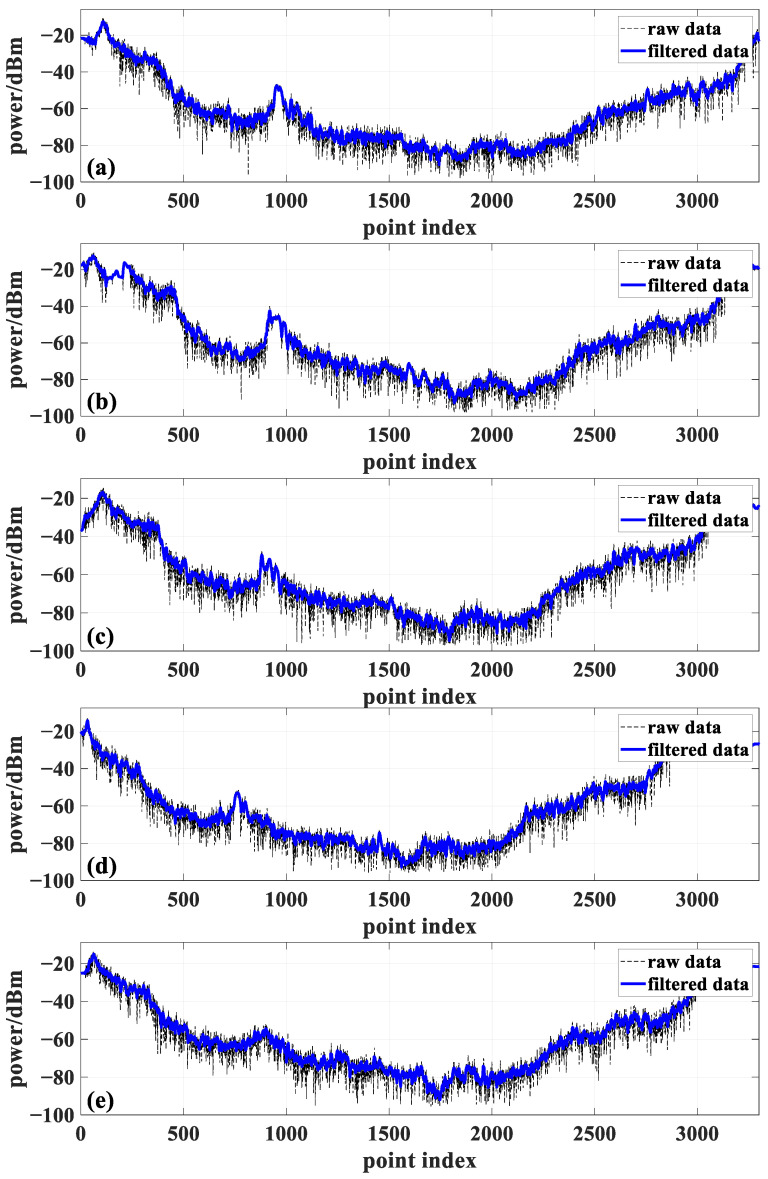
Raw power measurement data and power measurement data after applying the sliding filter at (**a**) 3 GHz frequency, (**b**) 3.6 GHz frequency, (**c**) 4 GHz frequency, (**d**) 5 GHz frequency, and (**e**) 5.9 GHz frequency.

Given the positional inaccuracies in the raw power measurement data, a dB-based sliding window method was applied to process the received data. This approach mitigates power fluctuations caused by phase variations due to minor positional errors. The sliding window size was set to five data points. Figure 10 presents the results after applying the sliding filter for the five frequency points, where the black line represents the raw measurement data, and the blue line represents the filtered data.

#### 4.2.2. Power Simulation and Verification

Both the algorithm presented in [39] and the algorithm proposed in this paper utilize a combination of three reflections and two diffractions for the calculations. Table 2 lists the material properties used in the algorithms, including the conductivity σ and the relative dielectric constant μ. In the measurement environment in this study, the building walls were composed of glass, the exterior wall decorations were made of aluminum, and the ground material was concrete.

**Figure 11:**
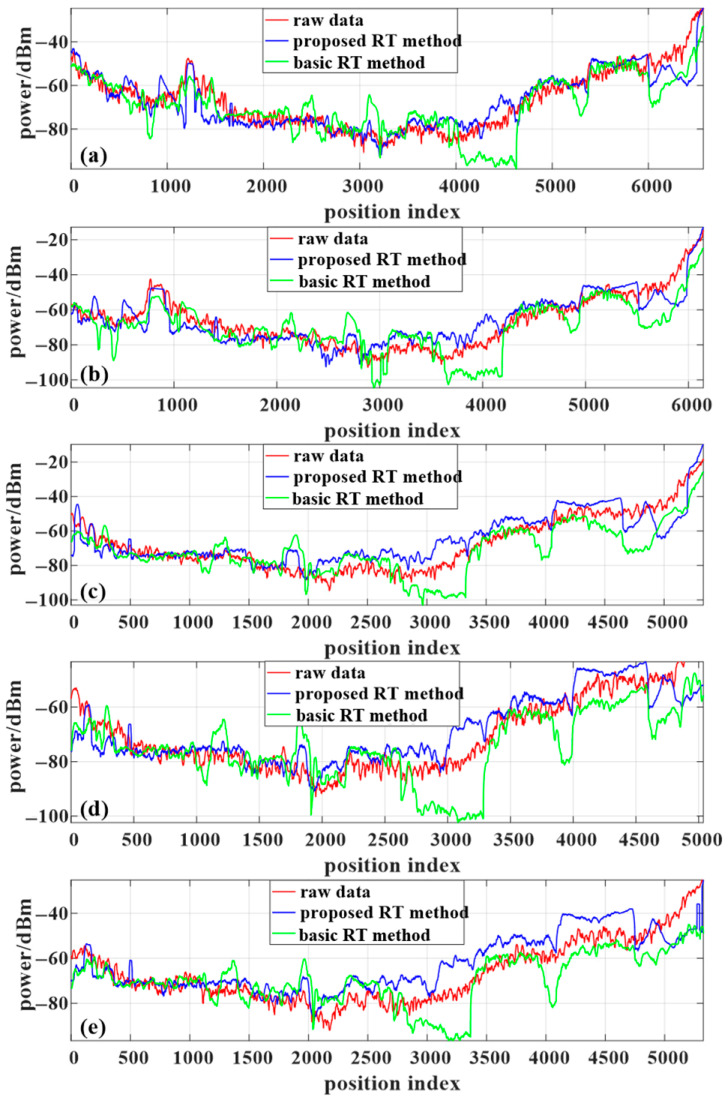
RSS predictions and measurements in the scenario at (**a**) 3 GHz frequency, (**b**) 3.6 GHz frequency, (**c**) 4 GHz frequency, (**d**) 5 GHz frequency, and (**e**) 5.9 GHz frequency. The basic RT method refers to the approach presented in [39].

The means and standard deviations of the comparison between the proposed algorithm and that in reference [39] against the measured data are summarized in Table 3. As illustrated by Figure 11 and Table 3, the proposed algorithm provides a notably superior performance in power prediction compared to the algorithm presented in [39]. For the initial 2500 data points, both algorithms exhibit strong agreement with the measured data. This is primarily due to the fact that, in regions with mild shadowing, the primary path energy is largely dominated by low-order reflections and diffractions, resulting in stable path search performance for both algorithms. However, under severe NLOS conditions (e.g., data points 4000–4600 for 3 GHz in Figure 11), the main path energy becomes more scattered, and the algorithm from reference [39] fails to capture certain multipath components, leading to a mismatch with the measured data and a significant loss of accuracy in deep shadowed areas. In contrast, the proposed algorithm is capable of identifying a greater number of multipath components even in such challenging deep shadow conditions, thereby achieving enhanced computational accuracy in NLOS scenarios.

#### 4.2.3. AOA Measurement Data Processing

**Figure 12:**
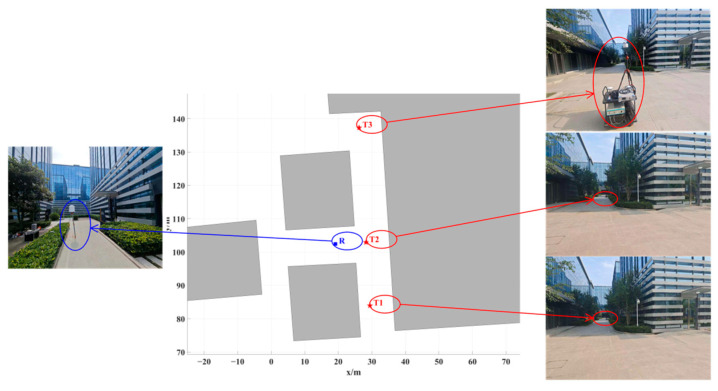
The angle measurement scenario and the positions of the NCTS (denoted by T1, T2, and T3) and sensor (denoted by R).

The angle measurement scenario was selected in a typical NLOS T-shaped street canyon within the park, as shown in Figure 12. The sensor was positioned in a canyon with a width of 12 m, while three source locations were placed in a canyon with a width of 9 m. T1 and T3 were located at the two ends of the canyon, both maintaining an NLOS condition with respect to R. T2 was placed at the intersection of the canyon, where it had a LOS condition with respect to R.

**Figure 13:**
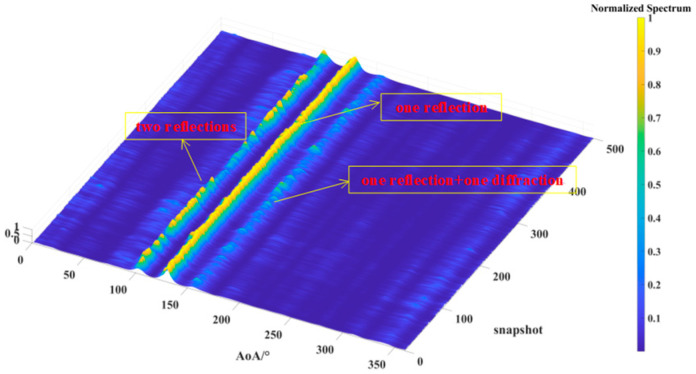
The AOA spectrum measured for the source located at T1.

**Figure 14:**
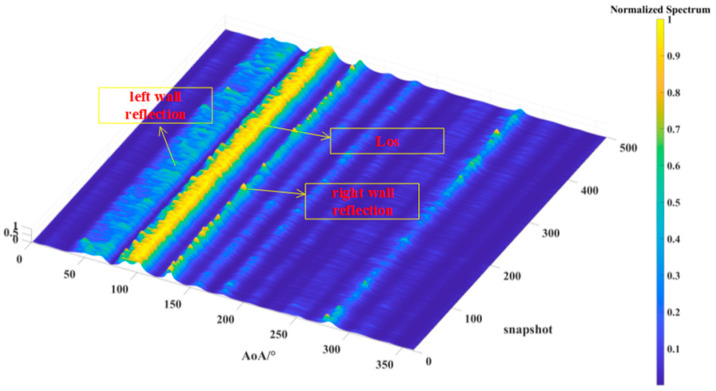
The AOA spectrum measured for the source located at T2.

**Figure 15:**
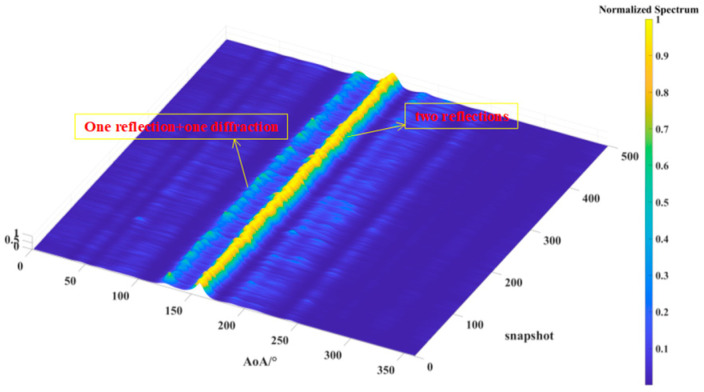
The AOA spectrum measured for the source located at T3.

Figure 13, Figure 14 and Figure 15 illustrate the results of 500 AOA measurements conducted at the three sites, T1, T2, and T3, at 5 GHz frequency. During the measurement process, both the direction-finding sensor and the source were maintained at a height of 1.85 m and remained stationary relative to each other. Table 4 summarizes the multipath angle power differences extracted from the measured AOA spectra at locations T1, T2, and T3.

#### 4.2.4. Localization Algorithm Verification

**Figure 16:**
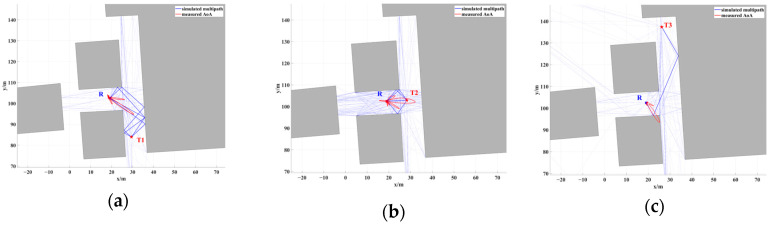
Comparison between measured AS and simulated multipath at (**a**) T1 position, (**b**) T2 position, and (**c**) T3 position.

Figure 13, Figure 14 and Figure 15 depict the multipath clusters with higher received power along with their corresponding propagation mechanisms. Figure 16 presents a comparison between the average AOA spectrum derived from 500 measurements and the results obtained using the proposed RT algorithm. The red curve represents the AOA spectrum received at point R, the solid blue line indicates multipath clusters with energy within 40 dB of the first arrival path, while the light blue line represents clusters with energy lower than 40 dB of the first path. Table 5 provides the differences between the root mean square (RMS) angular spread (AS) predicted by the proposed algorithm and the measured angular spread. Both the figures and tables indicate that the proposed algorithm demonstrates strong consistency with the measured AOA spectrum.

The accuracy of the proposed localization algorithm was evaluated and verified by combining the measured angles and power values listed in Table 4. Based on the proposed algorithm, the positioning errors for T1, T2, and T3 are documented in Table 6. All positioning errors are within a 1 m range, with an average error of approximately 0.69 m.

## 5. Efficiency and Performance Analysis of Localization Algorithm

### 5.1. Comparison of Localization Accuracy with Different AOA and RSSD Errors

**Figure 17:**
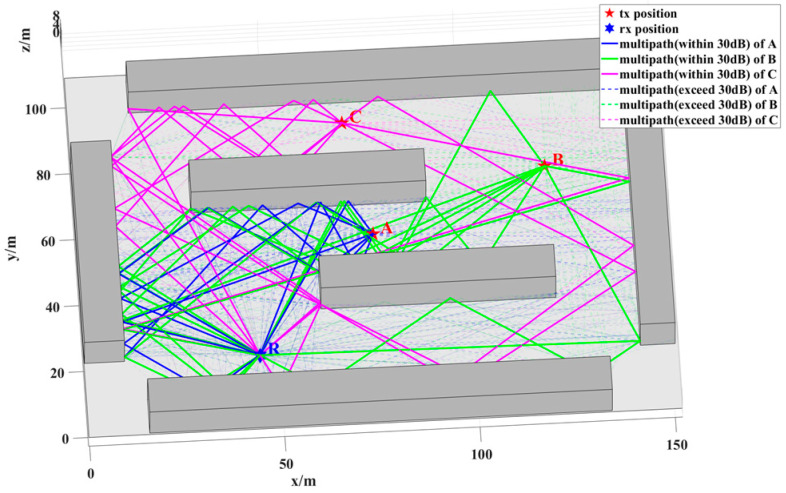
NCTS and sensor positions and a geometrical map of the scenario. The line segments represent the multipath between the source and the sensor, distinguished using different colors.

In this section, a representative NLOS scenario is employed to evaluate the localization accuracy of the proposed algorithm. The scenario consists of six buildings, each approximately 10 m in height, spanning an area of 110 m × 150 m. The sensor is located at point R with coordinates (45, 21), while three NCTSs, A, B, and C, are positioned at (76, 56), (121, 74), and (70, 90), respectively. Figure 17 depicts the simulation environment along with the locations of points R (receiver) and A, B, and C (transmitters). The three NCTSs (A, B, and C) are all in NLOS conditions relative to sensor R, with the degree of NLOS conditions ranked by severity. The multipath propagation between R and stations A, B, and C is shown in Figure 17, where solid lines represent multipath clusters with received power within 30 dB of the first path, and dashed lines denote clusters with received power more than 30 dB below the first path.

Single-station localization is performed for each of points A, B, and C. For point A, the received multipath is predominantly composed of paths involving up to three reflections. For point B, the received multipath consists mainly of paths involving up to four reflections and one diffraction. For point C, the received multipath comprises paths involving 2 to 4 reflections and 1 diffraction.

**Figure 18:**
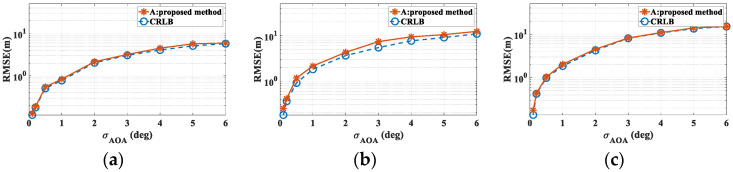
A comparison of the proposed localization algorithm’s accuracy with the CRLB. (**a**) The source at location A; (**b**) the source at location B; (**c**) the source at location C.

In the first round of simulations, the propagation mechanisms involved in localization include both reflection and diffraction. The sources are positioned at points A, B, and C, with errors due to received power being neglected. The AOA error is assumed to follow a zero-mean distribution with standard deviations ranging from 0.1° to 6°. For each error value, 10,000 simulations were performed. Figure 18 presents a comparison of the localization errors at points A, B, and C against the CRLB. The results demonstrate that the accuracy of the proposed RT-LBS algorithm approaches the CRLB. Moreover, considering the number of multipath components (MPCs) and the propagation distance between points A, B, C, and R, it can be deduced that point A exhibits the highest localization accuracy due to the larger number of MPCs and its relatively shorter propagation distance. In contrast, point C shows the lowest localization accuracy, attributable to fewer MPCs and a longer propagation distance.

**Figure 19:**
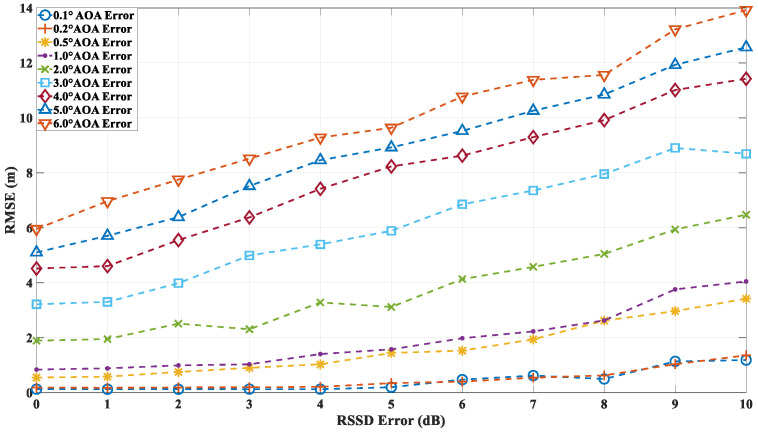
Localization error at point A with different AOA and RSSD errors.

**Figure 20:**
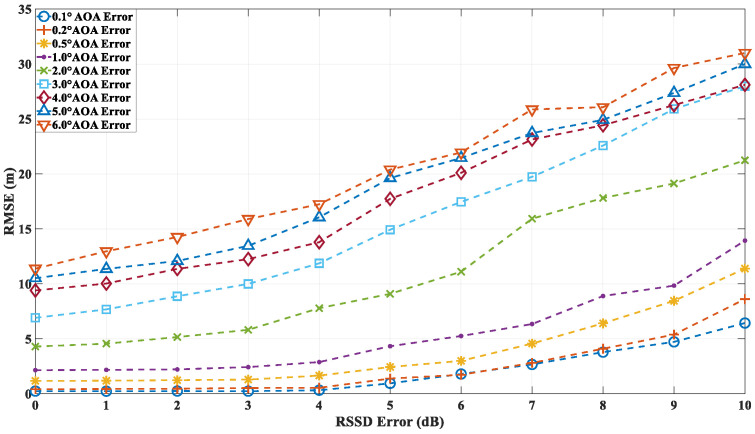
Localization error at point B with different AOA and RSSD errors.

**Figure 21:**
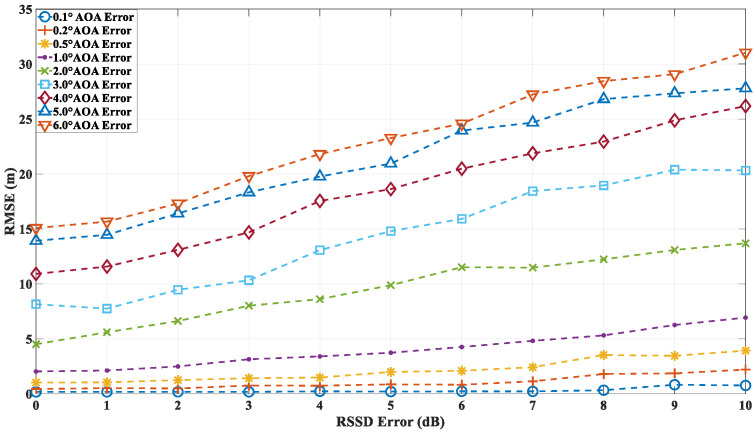
Localization error at point C with different AOA and RSSD errors.

In the second round of simulations, the localization accuracy of the algorithm is evaluated under the combined influence of both angle and power errors. The RMSE is calculated based on 10,000 independent simulations. As in the first simulation, the AOA error is modeled as a Gaussian random variable with zero mean and a standard deviation ranging from 0.1° to 6°. The RSS deviation, which directly affects the received multipath power, is also modeled as a Gaussian random variable with zero mean and a standard deviation between 0 dB and 10 dB. Figure 19 illustrates the localization accuracy at point A under different AOA and RSSD error conditions. The proposed algorithm demonstrates that, as the RSSD error increases, the localization accuracy is impacted to some extent. However, under low-AOA-error conditions, even with RSSD errors reaching up to 10 dB, the localization accuracy remains relatively high. In contrast, under high-AOA-error conditions, the localization accuracy degrades linearly with increasing RSSD error. Figure 20 and Figure 21 present the localization errors at points B and C, respectively, for varying AOA and RSSD errors.

The simulation results at points A, B, and C demonstrate that the proposed algorithm is capable of accurately localizing the source not only in environments with low-order reflections and diffractions (e.g., point A) but also in more complex scenarios involving higher-order reflections (e.g., points B and C). Given the prediction accuracy of the ray-tracing algorithm proposed in this study, which is approximately 6 dB, the localization accuracy at point A can be maintained within 2 m when the angle error is around 1°. Similarly, at points B and C, the accuracy remains within 5 m under the same conditions. These findings highlight the robustness of the proposed algorithm across a range of propagation environments and error conditions.

### 5.2. Localization Error Analysis Across the Entire Plane

In this section, a study of the localization error across the plane is conducted based on the scenario shown in Figure 17. The direction-finding sensor is placed at point R, while the NCTSs are uniformly distributed over a rectangular area (120 m × 80 m) with a spacing of 0.5 m. Both the sensor and the NCTSs are equipped with omnidirectional antennas. The power of the non-cooperative sources is set to 1.0 W, and the operating frequency is 5 GHz. A combination of up to four reflections and one diffraction is configured for the GS search.

As the proposed algorithm is fundamentally based on the ray-tracing technique, the accuracy of the localization process is directly contingent upon the precision of the ray-tracing algorithm. However, during planar localization, as the source position shifts, certain MPCs may emerge or disappear. This dynamic evolution of MPCs gives rise to regions characterized by significant multipath variability, where the multipath features of adjacent coordinate points exhibit substantial disparities. When the source is located within such regions of pronounced multipath fluctuations, the increase in angular error may lead to a corresponding escalation in localization error. This phenomenon, wherein rapid changes in multipath characteristics result in localization inaccuracies, is referred to as multipath fluctuation error (MFE).

Furthermore, multipath similarity constitutes another critical factor that can contribute to localization error, which is represented by the multipath similarity error (MSE). In this paper, multipath similarity is defined as the condition where the objects encountered at each propagation node across different MPCs are identical, and the AOA and power levels exhibit high degrees of similarity. In regions where multipath similarity prevails, the localization algorithm is likely to yield similar solutions, thereby linking the localization error to the extent of the similarity region.

Thus, in planar localization, both MFE and MSE emerge as significant sources of degradation in the accuracy of the localization algorithm. These factors underscore the challenges posed by complex multipath environments in achieving high-precision localization.

**Figure 22:**
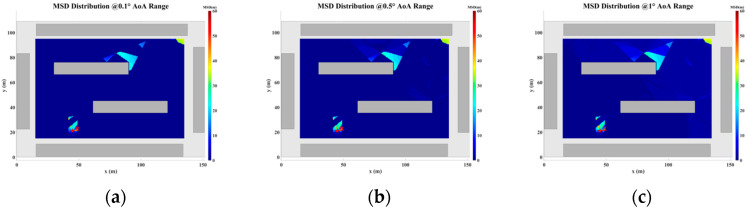

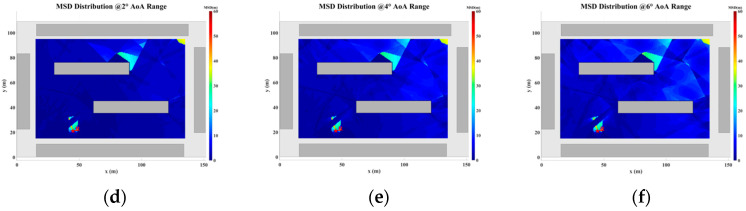
MSD distribution at (**a**) 0.1° AOA error, (**b**) 0.5°AOA error, (**c**) 1°AOA error, (**d**) 2°AOA error, (**e**) 4°AOA error, and (**f**) 6°AOA error.

To address the challenges posed by MFEs and MSEs, this paper introduces a displacement correction method based on a multipath similarity displacement matrix (MSDM). Figure 22a–f illustrate the MSDM under AOA error deviations ranging from 0.1° to 6°. Each element in these matrices represents the average adjacent multipath similarity distance (MSD) at the corresponding coordinate point. For instance, if the average MSD at point p is denoted by d, it can be inferred that the MPCs within a circular region centered at point p, with a radius of d, exhibit similar characteristics. As the angular deviation increases, the average adjacent MSD in the matrix also expands, indicating that the range of multipath similarity grows progressively larger. This relationship underscores the increasing spatial correlation of MPCs as angular errors rise.

**Figure 23:**
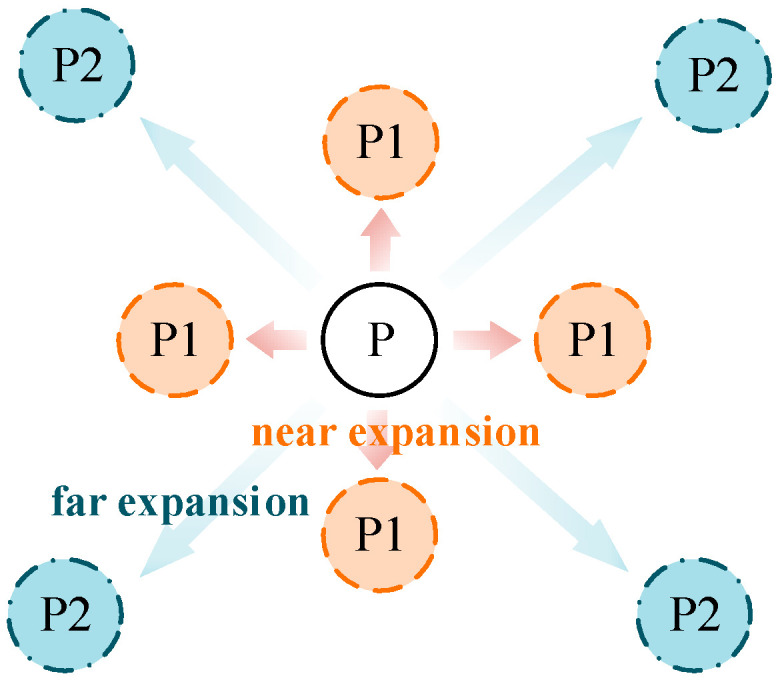
Schematic diagram of displacement compensation expansion method.

The displacement compensation expansion method expands the single coordinate solution in the GS cluster to nine coordinate solutions, as illustrated in Figure 23. To account for multipath fluctuation errors, the algorithm compensates by extending the solution by a minimum resolution distance around the coordinate point. For multipath similarity errors, the algorithm expands the solution by the average adjacent distance d from the corresponding matrix around the coordinate point, serving as a compensatory solution.

**Figure 24:**
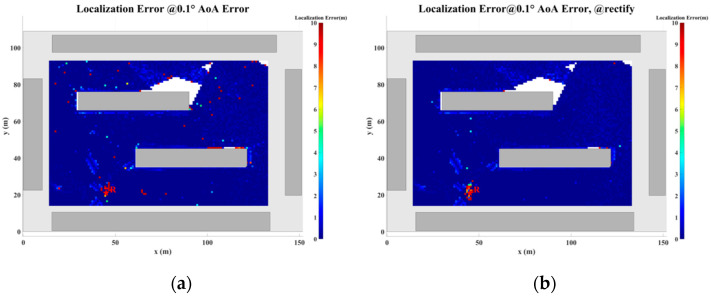
Planar Localization Error Distribution with 0.1° AOA error. (**a**) Original localization algorithm; (**b**) localization algorithm with MSDM.

**Figure 25:**
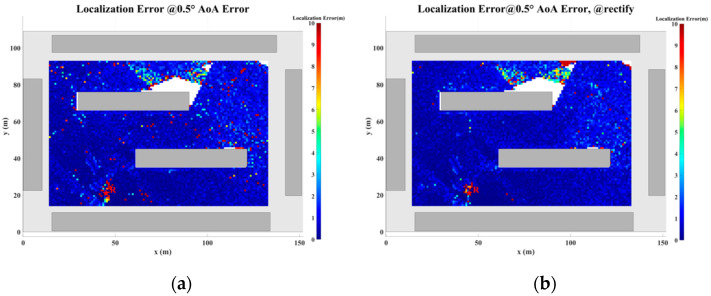
Planar Localization Error Distribution with 0.5° AOA error. (**a**) Original localization algorithm; (**b**) localization algorithm with MSDM.

**Figure 26:**
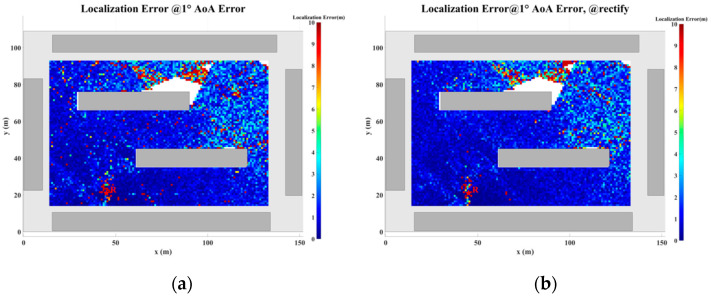
Planar Localization Error Distribution with 1° AOA error. (**a**) Original localization algorithm; (**b**) localization algorithm with MSDM.

**Figure 27:**
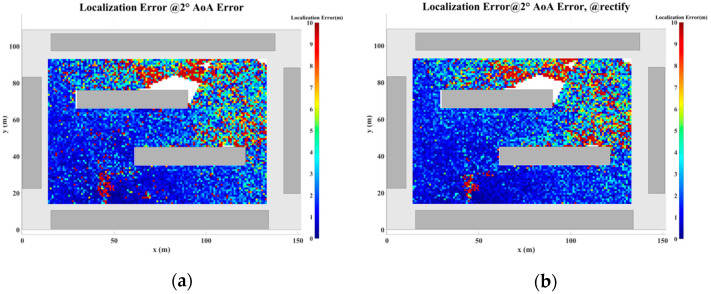
Planar Localization Error Distribution with 2° AOA error. (**a**) Original localization algorithm; (**b**) localization algorithm with MSDM.

**Figure 28:**
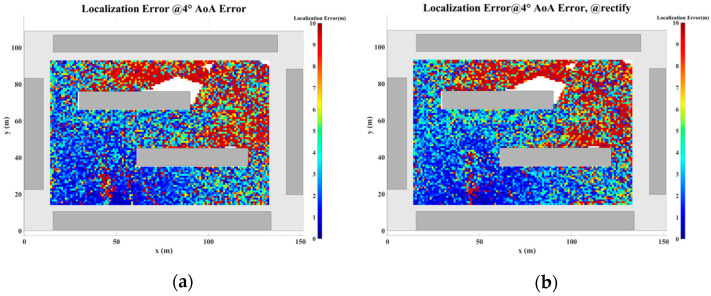
Planar Localization Error Distribution with 4° AOA error. (**a**) Original localization algorithm; (**b**) localization algorithm with MSDM.

**Figure 29:**
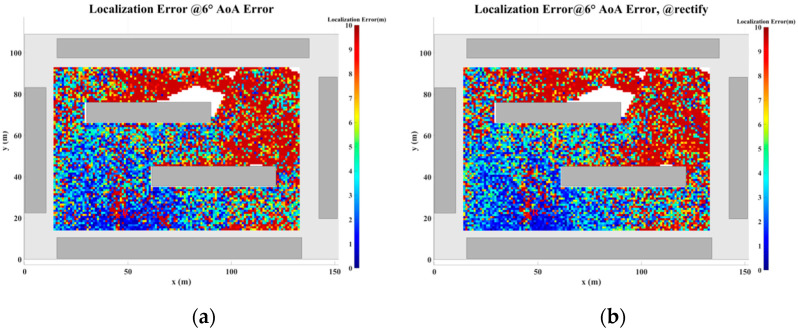
Planar Localization Error Distribution with 6° AOA error. (**a**) Original localization algorithm; (**b**) localization algorithm with MSDM.

Figure 24, Figure 25, Figure 26, Figure 27, Figure 28 and Figure 29 illustrate the planar localization accuracy distribution for both the original algorithm and the displacement correction algorithm. The optimized localization algorithm significantly improves accuracy, particularly at the shadow boundaries. The blank areas in the figures represent regions with no solution, primarily due to an insufficient number of multipath signals reaching these areas.

Table 7 summarizes the key performance metrics of the localization algorithm with the displacement correction method applied. The average localization accuracy of the algorithm improves by approximately 0.5 m, with the proportion of localization errors less than 10 m increasing by an average of 1.4%.

### 5.3. GPU-Accelerated Analysis of Localization Algorithm

**Figure 30:**
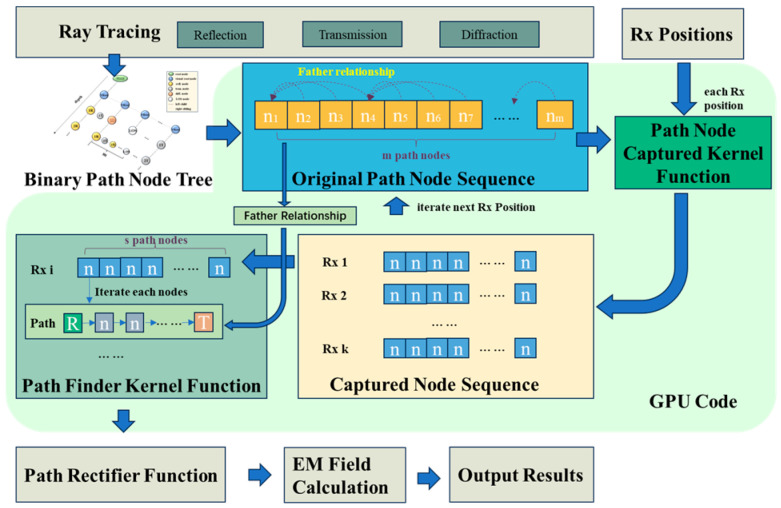
Schematic diagram of GPU acceleration algorithm.

The most computationally intensive component of the proposed localization algorithm lies in the path-finding process within the ray-tracing algorithm. Consequently, the acceleration method introduced in this work is specifically designed to optimize the path-finding procedure. As illustrated in Figure 30, the technical workflow of the proposed acceleration method proceeds as follows: First, the CPU performs a DFS of the binary tree structure, storing the resulting path node sequences in GPU memory. Next, the path node capture kernel function is employed to compute the valid path node sequences for the captured receiver points. Finally, the path-finding kernel function is invoked to search for the paths corresponding to each valid node and to apply the necessary corrections. This approach significantly enhances computational efficiency while maintaining path accuracy.

To assess the computational efficiency of the proposed algorithm, simulations were performed using a detailed electronic map of Xi’an, Shaanxi Province, China, extracted from OpenStreetMap. This dataset comprises 6189 buildings and more than 35,700 facets. The transmitter was positioned at the center of the map, elevated to a height of 200 m, with a transmission power set to 50 W and an operating frequency of 1 GHz. The receivers were placed 2 m above ground level and uniformly distributed across a rectangular area of 2 km × 2 km, with a spacing of 1 m between them. Both the transmitter and receivers employed vertically polarized omnidirectional antennas.

**Figure 31:**
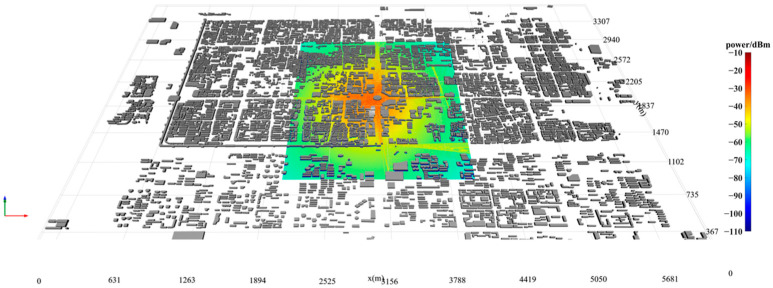
Power coverage map.

**Figure 32:**
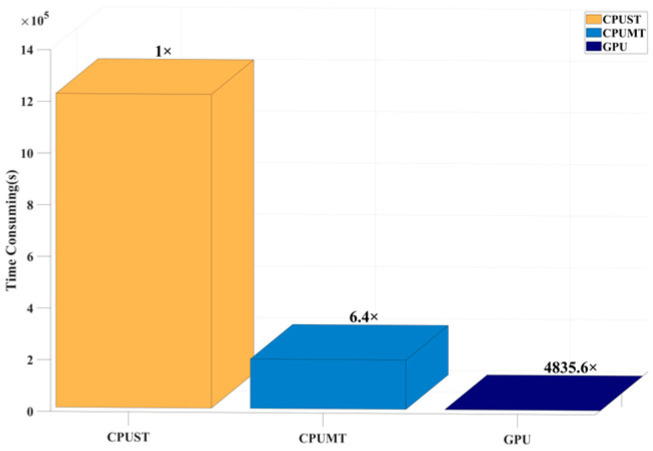
Efficiency comparison of different acceleration methods.

The proposed algorithm is fully implemented in C++/CUDA C++. Simulations were conducted on a desktop computer equipped with an Intel(R) Core(TM) i9-13900K CPU, an NVIDIA RTX 3090 GPU, and 128 GB of memory. To evaluate the computational efficiency of the GPU-accelerated algorithm, partial surface simulations were carried out for the scenario depicted in Figure 31, covering a total of 35,688 points. The simulation parameters were configured to allow up to three reflections and one diffraction. The simulation runtimes are presented in Figure 32 and Table 8, demonstrating that the GPU-based approach achieved a remarkable speedup of approximately 4835.6 times compared to the single-core CPU implementation, with an average computation time of 7 milliseconds per receiving point.

## 6. Discussion and Future Work

Based on the series of empirical measurements and simulation tests presented in this paper, the effectiveness and robustness of the proposed NLOS localization algorithm have been demonstrated. However, we have identified two primary limitations of the proposed algorithm that warrant further exploration in future research:

(1) The geometric modeling in this study utilizes high-precision LiDAR point cloud data with an accuracy of up to 5 cm. However, this precision is negligible compared to errors caused by multipath propagation, meaning potential biases from building structure inaccuracies in digital maps are not accounted for here. Even with lower-accuracy maps, such as those sourced from platforms like OpenStreetMap, the prediction accuracy of the ray-tracing algorithm remains acceptable [45]. However, in practical applications, such high-precision data are rarely available. Therefore, future research should address errors from low-accuracy digital maps to enhance the algorithm’s adaptability and performance in real-world scenarios.
(2) The proposed localization algorithm relies on RT, with its accuracy directly affecting localization performance. However, the current model overlooks the impact of vegetation penetration, reducing prediction accuracy in vegetated areas.

Furthermore, we suggest the following areas as key focal points for future research:

(1) Given the above limitation regarding vegetation, future work should integrate a vegetation penetration model to improve localization accuracy in such environments.
(2) The localization algorithm was evaluated in urban NLOS scenarios, but NLOS conditions are often more prominent indoors, suggesting the need for further investigation. The current 2D RT algorithm also struggles with propagation mechanisms involving floors and ceilings in enclosed spaces. Developing a 3D localization algorithm could significantly improve accuracy in such environments.

## 7. Conclusions

This paper proposes a single-station localization method that integrates an RT algorithm designed explicitly for NLOS environments. In complex NLOS scenarios, signals emitted by the source undergo multipath propagation before reaching the sensor. The proposed method exploits the AOA and the received power differences to construct a GS set with initial weights based on a GS filtering rule. Each GS corresponds to a nonlinear equation derived from the AOA. By incorporating the IRLS method, the algorithm achieves high-accuracy target localization. Simulations and experiments demonstrate that positioning accuracy can achieve meter-level or even sub-meter-level precision, closely approaching the CRLB. A key advantage of the proposed approach is that it relies solely on the AOA spectrum from a single station, making it highly practical for engineering applications.

## Figures and Tables

**Table 1:** Configuration of measurement system.

Configuration	Description
Carrier frequency	3 GHz, 3.6 GHz, 4 GHz, 5 GHz, 5.9 GHz
Signal constitution	CW
Speed of Rx	5 km/h
Transmission power	38 dBm
Tx/Rx antenna	Vertically polarized omnidirectional antenna
Tx/Rx antenna gain	2.7 dB
Tx position	(−0.79, 5.656, 1.85) m
Route distance	640 m–700 m
Power measurements per second	20
RTK location records per second	20

**Table 2:** Electrical material data in the environment [44].

Material	Scenario Part	σ (S/m)	μ
Glass	Wall	9.82 × 10^−3^	6.27
Aluminum	Wall decorations	3.5 × 10^7^	7.6
Concrete	Ground	5.71 × 10^−2^	5.31

**Table 3:** Error comparison between predicted and measured results.

Frequency (GHz)	Mean (dB)		Standard Deviation (dB)	
	RT	Proposed RT	RT	Proposed RT
3.0	2.40	3.25	8.0	6.39
3.6	3.38	−1.72	8.94	7.37
4.0	1.82	0.89	9.05	7.72
5.0	−2.62	0.61	8.93	6.84
5.9	−3.89	2.8	8.53	7.27

**Table 4:** Measured AOAs and RSSDs.

Index	T1		T2		T3	
	AOA	RSSD	AOA	RSSD	AOA	RSSD
1	129°	0 dB	90°	0 dB	126°	0 dB
2	38°	−3.4 dB	42°	−6.8 dB	148°	−22.3 dB
3	123°	−6.6 dB	51°	−26.3 dB	79°	−23.5 dB
4	50°	−17.1 dB	161°	−33.7 dB	229°	−26.3 dB
5	126°	−25.3 dB	-	-	-	-

**Table 5:** Angular spread error comparison between simulated and measured results.

Source Name	AS Measured	AS Simulated	Absolute Error
T1	40.24°	42.82°	2.58°
T2	18.23°	21.62°	3.39°
T3	6.09°	3.56°	2.53°

**Table 6:** The absolute localization error of the proposed algorithm.

Source Name	Position Measured	Position Estimated	Absolute Error
T1	(29.44, 83.94) m	(30.04, 83.69) m	0.64 m
T2	(28.25, 102.92) m	(28.44, 103.24) m	0.37 m
T3	(26.21, 137.33) m	(25.25, 122.85) m	1.07 m

**Table 7:** A comparison of the mean error and localization accuracy within 10 m between the original localization algorithm and the algorithm with the MSDM applied.

AOA Error	Mean Error		Localization Error Rate (<10 m)	
	Original	with MSDM	Original	with MSDM
0.1°	0.367 m	0.234 m	99.36%	99.82%
0.5°	1.120 m	0.956 m	98.51%	99.19%
1.0°	2.053 m	1.840 m	96.81%	97.88%
2.0°	4.032 m	3.495 m	92.71%	94.17%
4.0°	7.670 m	6.950 m	81.54%	83.38%
6.0°	11.036 m	9.780 m	71.16%	74.08%

**Table 8:** Efficiency test of algorithm acceleration method.

Acceleration Type	Time Consumed	Speed Up
CPU Single Thread	1,214,122.13 s	1×
CPU Multi-Thread	190,899.7 s	6.4×
GPU	251.08 s	4835.6×

## Data Availability

The data that support the findings of this study are available by contacting the corresponding author.
